# Placental hypoxia-regulating network in relation to birth weight and ponderal index: the ENVIR*ON*AGE Birth Cohort Study

**DOI:** 10.1186/s12967-017-1375-5

**Published:** 2018-01-10

**Authors:** Karen Vrijens, Maria Tsamou, Narjes Madhloum, Wilfried Gyselaers, Tim S. Nawrot

**Affiliations:** 1 Center for Environmental Sciences, Molecular & Environmental Epidemiology, Hasselt University, Agoralaan Building D, 3590 Diepenbeek, Belgium; 2 Department of Obstetrics, East-Limburg Hospital, 3600 Genk, Belgium; 3 Department of Public Health, Environment & Health Unit, Leuven University (KU Leuven), 3000 Louvain, Belgium

**Keywords:** Birth weight, Gene expression, HIF1α, Hypoxia, Placenta

## Abstract

**Background:**

HIF1α, miR-210 and its downstream targets ISCU, COX-10, RAD52 and PTEN are all part of the placental hypoxia-responsive network. Tight regulation of this network is required to prevent development of maternal–fetal complications such as fetal growth restriction. HIF1α expression is increased in preeclamptic placentae, but little is known about its association with birth weight in normal pregnancies.

**Methods:**

We measured placental levels of *HIF1α*, miR-20a, miR-210, *ISCU*, *COX*-*10*, *RAD52* and *PTEN* in 206 mother–newborn pairs of the ENVIR*ON*AGE birth cohort.

**Results:**

Placental *HIF1α* gene expression was inversely associated with the ponderal index (PI): for a doubling in placental *HIF1α* expression, PI decreased by 6.7% (95% confidence interval [CI] 1.3 to 12.0%, p = 0.01). Placental *RAD52* expression also displayed an inverse association with PI, a doubling in gene expression was associated with a 6.2% (CI 0.2 to 12.1% p = 0.04) decrease in PI. As for birth weight, we observed a significant association with placental miR-20a expression only in boys, where a doubling in miR-20a expression is associated with a 54.2 g (CI 0.6 to 108 g, p = 0.05) increase in birth weight.

**Conclusions:**

The decrease in fetal growth associated with expression of hypoxia-network members *HIF1a, RAD52* and miR-20a indicates that this network is important in potential intrauterine insults.

## Background

**Fig. 1:**
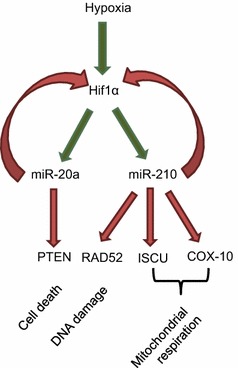
Hypoxia-responsive network in the placenta. miR-20a and miR-210 expression is upregulated via HIF1α. These miRNAs in turn regulate a broad spectrum of genes under hypoxia, including genes involved in mitochondrial biogenesis (*ISCU*), the respiratory chain (*COX10*), DNA double strand break repair (*RAD52*) and cell death (*PTEN*). miR-20a and miR-210 have both been shown to target *HIF1α* itself as well

Fetal growth is dependent on genetic, placental and maternal factors. The fetus is thought to have an inherent growth potential that, under normal circumstances, results in a healthy newborn of appropriate size. The maternal–placental–fetal units act in harmony to provide the needs of the fetus while supporting the physiologic changes of the mother. The most common cause of low birth weight (LBW) at term in Western societies is placental insufficiency [1]. LBW infants have a 10–20-fold increased risk of dying in the perinatal period [2] and are at increased risk of developing chronic diseases including type 2 diabetes, hypertension and heart disease in later life [3]. Fetal growth restriction is the second leading cause of perinatal morbidity and mortality [4]. The ponderal index has been installed as an indicator of fetal growth particularly able to identify normal weight newborns (birth weight above 2500 g) with higher probability to develop disease such as hypoglycemia and hyperbilirubinemia (lower ponderal index) [5]. Placental development is dependent on tight regulation of oxygen tension [6]. Hypoxia-inducible factor 1α (HIF1α) plays an important role in control of placental oxygenation. Normal pregnancy is hallmarked by a state of oxidative stress, in which placental mitochondria generate reactive oxygen species [7]. Feto-placental hypoxia, hallmarked by increased levels of *HIF1α*, has been associated with development of preeclampsia [8]. A placental mRNA/miRNA network involving microRNA (miR)-210, iron sulfur-cluster assembly enzyme (ISCU) and HIF1α was proposed to regulate mitochondrial function during preeclampsia [9]. HIF1α is at the core of the hypoxia-responsive network able to target mRNAs and miRNAs executing diverse cellular functions [10]. HIF1α not only induces expression of miRNAs-20a and -210, these miRNAs in turn were shown to downregulate *HIF1α* [11, 12]. Preeclampsia is one of the most common pregnancy complications and it is believed that an inadequate blood flow in the placenta is responsible for this [13]. Preeclampsia could lead to fetal growth restriction. Fetal growth restriction is associated with increased fetal and perinatal morbidity and mortality, even in the absence of preeclampsia [14]. Little is known on the relation between *HIF1α* expression in placental tissue from normal term pregnancies and fetal growth. As tight regulation of oxygen levels is essential for proper fetal-placental development, we hypothesize placental expression levels of the hypoxia-responsive network are associated with measures of fetal growth in normal pregnancies. We analyzed expression of 7 members from the hypoxia-regulation network in placental tissue (Fig. 1), by examining miR-20a and miR-210 and mRNA *HIF1α*, cytochrome C oxidase-10 (*COX*-*10)*, *ISCU*, phosphatase and tensin homolog (*PTEN)*, and DNA repair protein *RAD52* expression levels.

## Methods

### Study design and population

From the ongoing population-based Birth Cohort Study ENVIR*ON*AGE (ENVIRonmental influence *ON* AGEing), 206 mother–child pairs recruited between September 2011 and January 2014 at East Limburg Hospital (Genk, Belgium) were included in the current study. Inclusion criteria were singleton pregnancy and ability to fill out questionnaires in Dutch. The overall participation rate of eligible mothers was 61% [15] and we previously demonstrated the cohort represents birth in Flanders well [16].

Study approval was obtained from the ethics committees of East Limburg Hospital and Hasselt University and has been carried out according to the declaration of Helsinki. Written informed consent was obtained from the mothers prior to participation. Information on maternal age, smoking behavior (mother and co-residents), ethnicity, pre-pregnancy BMI and parity was obtained through questionnaires. Perinatal parameters such as newborn’s sex, birth date, birth weight and gestational age were collected from birth records. Ponderal index (PI) was calculated according to Rohrer’s formula, PI = (birth weight in grams/birth length in cm^3^) × 100.

### Sample collection

Placentas were collected within 10 min after delivery and frozen at − 20 °C. Placentas were thawed minimally to obtain tissues biopsies for RNA extraction. To minimize the impact of within-placenta variability, biopsies were taken 1–1.5 cm below the chorio-amniotic membrane at 4 fixed locations across the placenta and stored overnight in RNA later and then preserved at − 20 °C until use.

### RNA extraction

Total RNA and miRNA were extracted from pooled placenta biopsies using the miRNeasy mini kit (Qiagen, KJ Venlo, the Netherlands) according to the manufacturer’s instructions. Pooled placental samples from 4 biopsies were used to minimize intra-placental variation. Quality control of the extracted total RNA and miRNA was assessed by spectrophotometry (Nanodrop ND-1000; Isogen Life Science, De Meern, the Netherlands) and, for a random subset of the samples, 2100 Bionanalyzer (Applied Biosystems, Foster City, CA). The average (± SD) yield of total RNA per placenta was 4.3 (± 1.2) µg with average A_260/280_ and A_260/230_ ratio of 1.96 (± 0.02) 1.85 (± 0.18), respectively. DNase treatment was performed on extracted RNA samples according to the manufacturer’s instructions (Turbo DNA-free kit, Ambion, Life Technologies, Diegem, Belgium). Isolated RNA was stored at − 80 °C until further use. We previously reported [17] the variability within the four individual biopsies in a subset of ten placental tissues. Within the four biopsies of each placenta the Ct values for miRNA expression varied between 2 and 9% (CV). We therefore opted to use pooled samples from the 4 biopsies to minimize inter-placental differences.

### Gene expression analysis

A maximum amount of 3 μg of total RNA was reverse transcribed into cDNA for gene expression analysis using the GoScript Reverse Transcription System (Promega, Madison, WI, USA) on a Veriti 96 well Thermal cycler (TC-5000, Techne, Burlington, NJ, USA). cDNA was stored at − 20 °C until use. A quantitative real-time polymerase chain reaction (qPCR) was set up by adding 2 μL of a 10 ng/μL dilution of cDNA together with TaqMan Fast Advanced Master Mix (Life Technologies, Foster City, CA, USA) and PrimeTimeTM assay (Integrated DNA Technologies, Coralville, IA, USA) for target mRNAs *HIF1α*, *RAD52*, *COX*-*10*, *ISCU* and *PTEN* in a final reaction volume of 10 μL. Standard cycling conditions were used to analyze samples in a 7900HT Fast Real-Time PCR system (Life Technologies, Foster City, CA, USA). Cq values were collected with SDS2.3 software. MIQE guidelines were taken into account [18]. Amplification efficiencies were between 90 and 110% for all assays. Raw data were processed to normalized relative gene expression values with qBase plus software (Biogazelle, Zwijnaarde, Belgium) using *IPO8*, *POLR2A*, *UBC*, *GAPDH* as reference genes for data normalization. Technical replicates were included when the difference in Cq value was < 0.5.

### miRNA expression analysis

For analysis of miR-20a and miR-210 expression, RNA was reverse transcribed using the Megaplex reverse transcription (RT) stemloop primer pool A (Applied Biosystems, Foster City, CA) according to the manufacturer’s protocol. Briefly, 375 ng total RNA was reverse transcribed as follows: 2 min at 16 °C, 1 min at 42 °C and 1 min at 50 °C, for 40 cycles (Thermocycler PCR, Techne, Staffordshire, UK). Afterwards, cDNA was stored at − 20 °C for a maximum of 1 week until qRT-PCR measurements were performed. miRNA qRT-PCR analysis was performed using Taqman miRNA assays (Applied Biosystems, Foster City, CA), according to the manufacturer’s protocol. An input of 5 ng cDNA was used for PCR reactions, which were run on a 7900HT Fast Real-Time PCR System (Applied Biosystems, Foster City, CA), as follows: a polymerase activation for 2 min at 50 °C, a denaturation step for 10 min at 95 °C and an anneal/extension step (40 cycles) for 15 s at 95 °C and for 1 min at 60 °C. For normalization the endogenous control RNU6 was used. In order to minimize the technical variation between the different runs of the same miRNA assay, inter-run calibrators (IRCs) were used. Cq values were collected with SDS 2.3 software and the relative miRNA expression was calculated using the 2^−ΔΔ^Cq method (qBase plus; Biogazelle, Belgium). All samples were analyzed in triplicate. Replicates were included when the ΔCq was < 0.5.

### Statistical analysis

SAS software (Version 9.3 SAS Institute, Cary, NC, USA) was used for statistical analysis. The relative quantities of mRNA expression were log-transformed because of their non-normal distribution. The collected data are presented as categorical data with frequencies (%) or numbers and as continuous data with mean (± SD). The association between relative placental mRNA/miRNA expression at birth and birth weight or ponderal index was assessed using a linear regression model. The model was corrected for the following covariates: newborn’s gender, ethnicity (European and non-European) and gestational age (weeks), maternal age (years), smoking status (never-smoker, past-smoker or current-smoker), educational status (low, middle or high) and parity (1, 2 or ≥ 3).

## Results

### General characteristics of the study population

Detailed maternal and newborn characteristics are shown in Table 1. Maternal age averaged (± SD) 29.3 ± 4.4 years. 69.9% of the population never smoked and 56.2% had a high educational level. The average maternal pre-gestational BMI was 24.3 ± 4.7 kg/m^2^. 53.7% of the newborns were female and 90.2% were of European decent. Gestational age averaged 39.2 weeks and ranged from 36 to 41 weeks. Mean ponderal index was 2.68 (± 0.29) g/cm^3^ and birth weight 3431 (± 447) g. Half (50.5%) of the newborns were their mothers’ first child and 2.9% were delivered by Caesarian-section (C-section).

**Table 1:** Demographic characteristics of the study population (n = 206)

Characteristics	Mean ± SD/Frequency (%)	
	Boys (n = 96)	Girls (n = 110)
Maternal		
Age, years	29.3 ± 4.4	29.5 ± 4.4
Pre-gestational BMI, kg/m^2^	24.6 ± 4.6	24.2 ± 5.2
Smoking status		
Never-smoker	64 (66.7)	79 (72.2)
Past-smoker	16 (16.7)	18 (16.5)
Current-smoker	16 (16.7)	13 (11.3)
Parity		
1	47 (49.0)	57 (51.8)
2	37 (38.5)	45 (40.9)
≥ 3	12 (12.5)	8 (7.3)
Education		
Low	13 (13.8)	10 (9.2)
Middle	37 (39.4)	29 (26.6)
High	44 (46.8)	71 (64.2)
Newborn		
Gestational age, weeks	39.2 ± 1.4	39.3 ± 1.2
Birth weight, g	3483 ± 483	3385 ± 410
Ponderal index, kg/m^3^ × 100	2.63 ± 0.24	2.72 ± 0.24
Ethnicity		
European-Caucasian	85 (88.5)	101 (91.74)
Non-European	11 (11.5)	9 (8.26)
C-section	3 (3.1)	3 (2.6)

### Placental expression of hypoxia-related factors in association with birth weight and ponderal index

**Fig. 2:**
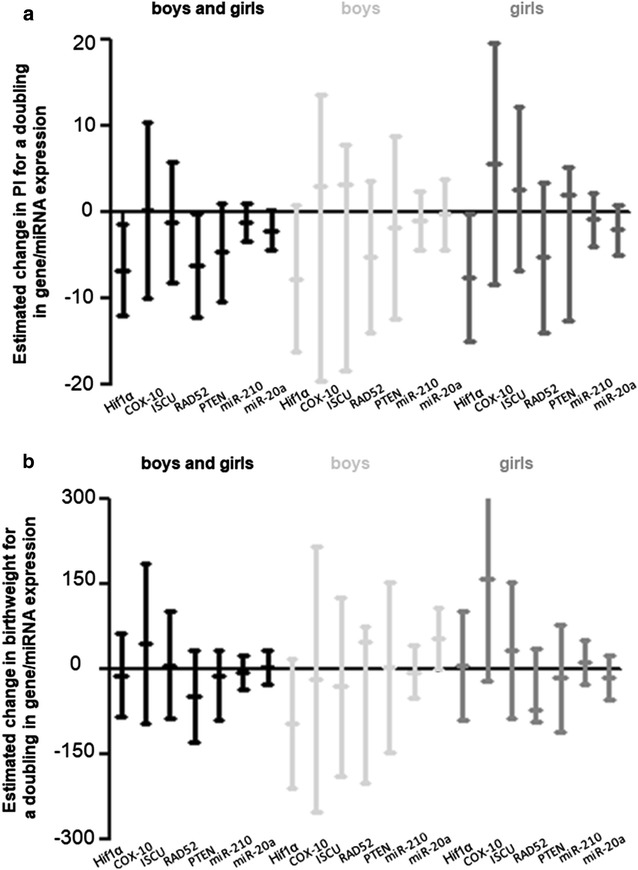
Association between ponderal index, birthweight and hypoxia-responsive network expression in placental tissue. Models were adjusted for newborns’ ethnicity, gestational age, maternal age, smoking and educational status of the mother, maternal pre-pregnancy BMI, and parity. Analysis in girls and boys combined was furthermore adjusted for newborns’ gender. Estimates are presented as an absolute change in PI (**a**) or birthweight (**b**) for a doubling in gene or miRNA expression

Expression of the placental hypoxia network was negatively associated with the ponderal index. Placental *HIF1α* gene expression was inversely associated with PI: for a doubling in placental *HIF1α* expression, PI decreased by 6.7% (95% confidence interval [CI] 1.3 to 12.0%, p = 0.01) when analyzing both sexes together, with similar estimates in boys and girls. Placental *RAD52* expression also displayed an inverse association with PI for boys and girls, a doubling in gene expression was associated with a 6.2% (CI 0.2 to 12.1% p = 0.04) decrease in PI. No significant associations with the other analyzed hypoxia-network genes and ponderal index could be observed, although there was a trend for a negative association between PI and miR-20a expression in girls and boys, where a doubling in gene expression was associated with a 2.08% (CI − 0.2 to 4.4%, p = 0.08) decrease in PI (Table 2, Fig. 2).

**Table 2:** Changes in relative placental mRNA and miRNA expression associated with ponderal index and birth weight, for a doubling in gene expression

Gene	Ponderal index		Birth weight	
	% change (95% CI)	p-value	% change (95% CI)	p-value
Girls and boys (n = 206)				
HIF1α	− 6.68 (− 12.01, − 1.34)	0.01	− 10.79 (− 83.67, 62.09)	0.77
COX-10	0.26 (− 9.96, 10.49)	0.96	45.70 (− 94.56, 185.97)	0.52
ISCU	− 1.20 (− 8.18, 5.78)	0.73	7.58 (− 86.83, 101.99)	0.87
RAD52	− 6.16 (− 12.10, − 0.22)	0.04	− 47.73 (− 127.70, 32.24)	0.24
PTEN	− 4.57 (− 10.27, 1.13)	0.12	− 11.20 (− 88.94, 32.24)	0.78
miR-210	− 1.18 (− 3.36, 0.99)	0.28	− 5.42 (− 34.84, 24.00)	0.72
miR-20a	− 2.08 (− 4.37, 0.22)	0.08	3.52 (− 27.55, 34.59)	0.82
Boys (n = 96)				
HIF1α	− 7.67 (− 16.18, 0.83)	0.08	− 96.16 (− 210.5, 18.17)	0.10
COX-10	3.03 (− 19.66, 13.59)	0.72	− 16.74 (− 250.22, 216.74)	0.89
ISCU	7.81 (− 18.33, 3.21)	0.16	− 30.88 (− 188.06, 126.31)	0.70
RAD52	− 5.14 (− 13.98, 3.70)	0.25	76.71 (− 201.02, 47.59)	0.22
PTEN	− 1.77 (− 12.33, 8.80)	0.74	3.01 (− 146.07, 152.09)	0.97
miR-210	− 0.93 (− 4.38, 2.51)	0.59	− 4.56 (− 51.57, 42.45)	0.85
miR-20a	− 0.25 (− 4.43, 3.93)	0.91	54.19 (0.57, 107.82)	0.05
Girls (n = 110)				
HIF1α	− 7.56 (− 14.97, − 0.14)	0.05	7.15 (− 88.27, 102.58)	0.88
COX-10	5.54 (− 8.46, 19.55)	0.43	160.21 (− 20.17, 340.59)	0.08
ISCU	2.73 (− 6.81, 12.27)	0.57	33.04 (− 87.12, 153.19)	0.59
RAD52	− 5.19 (− 13.91, 3.53)	0.24	− 72.35 (− 181.64, 36.94)	0.19
PTEN	5.22 (− 12.56, 2.11)	0.16	− 15.45 (− 110.14, 79.25)	0.75
miR-210	− 0.86 (− 3.90, 2.18)	0.58	11.27 (− 28.00, 50.54)	0.57
miR-20a	− 2.06 (− 5.02, 0.91)	0.17	− 15.46 (− 53.97, 23.04)	0.43

Estimated changes are shown with their 95% confidence intervals and p-values. Estimates were adjusted for newborns’ ethnicity, gestational age, maternal age, smoking and educational status of the mother, maternal pre-pregnancy BMI, and parity. Analysis in girls and boys combined was furthermore adjusted for newborns’ gender.

As for birth weight, we observed a significant association with placental miR-20a expression only in boys, where a doubling in *miR*-*20a* expression is associated with a 54.2 g higher birth weight (CI 0.6 to 108 g, p = 0.05). Finally, a trend towards a negative association between *COX*-*10* expression in girls and birth weight was observed, where a doubling in gene expression is associated with a 160 g increase (CI − 20.2 to 340.6, p = 0.08) in birth weight (Table 2). All analyses were corrected for newborns’ ethnicity, gestational age, maternal age, smoking and educational status of the mother, maternal pre-pregnancy BMI, and parity.

We tested the interaction terms of newborns parity and expression of hypoxia-network genes and microRNAs expression on birthweight and the ponderal index. Only the interaction term for RAD52 expression*parity was significant for birthweight (p = 0.036). When analyzing the association between gene/miRNA expression and birthweight subdivided by parity, we did not found any significant associations.

## Discussion

We here report on the expression of the placental hypoxia-responsive network involving HIF1α and several of its target genes/miRNAs and its association with fetal growth parameters. The relationship between low birth weight or low ponderal index and chronic disease has been established in several publications [5, 19, 20]. As such, an inverse relationship between birth weight and blood pressure in childhood was already observed in the 1980s by David Barker [21], which can lead to development of cardiovascular disease (reviewed in [22]).

Regulation of oxygen tension is tightly controlled throughout gestation, and follows a distinct pattern. During the first trimester, the placenta develops under low oxygen tension, a physiologic form of hypoxia, oxygen tension, as well as HIF1α expression, increase towards the end of the first trimester (week 11 of gestation) [23, 24]. From this point onwards, the placenta is well-oxygenated throughout the remainder of gestation and a physiologic state of normoxia exists. If placental oxygen tension drops towards the end of pregnancy, a hypoxic or ischemic condition arises that can be detrimental for both mother and fetus. HIF1α expression achieves two peaks during the first trimester, one around week 7–10 of gestation, another one around week 14–18. Levels drop much lower around the time of birth [24]. PTEN expression on the other hand was reported higher during the first trimester pregnancy as compared to any time in the normal menstrual cycle [25]. PTEN expression of villous trophoblasts was decreasing as the pregnancy advanced. PTEN expression decreased parallel to the development of placenta. Expression of PTEN in decidual cells was significantly stronger in placental tissues of spontaneous abortion than placental tissues from normal pregnancies at the first and third trimester [26]). miR-210 expression remains at fairly constant levels throughout healthy pregnancies, whereas its expression is increased in pregnancies complicated by preeclampsia between 26 and 38 weeks of gestation, this association disappears after 38 weeks of gestation [27].

We observed significant negative associations between placental *HIF1α*, *RAD52* and the ponderal index in newborn girls and boys. Moreover, we found a significant positive association between placental expression of miR-20a and birth weight in newborn boys only. Our findings support the importance of expression of these genes in the placenta for normal fetal growth. Decreased expression of *HIF1α* has previously been reported in placental tissue from pregnancies complicated by preeclampsia compared to normal pregnancies [8, 28]. Preeclampsia is hallmarked by hypertension and proteinuria and is associated with significant maternal morbidity and mortality [29]. Preeclampsia is characterized by placental hypoxia and/or ischemia, excessive oxidative stress, in association with endothelial dysfunction [30]. Whether *HIF1α* expression is associated with intrauterine growth restriction (IUGR) is not clear. Some studies report no detected differences in placental *HIF1α* expression between IUGR babies versus babies born from normal singleton pregnancies [31], whereas others have reported increased expression of *HIF1α* in maternal plasma [32]. Zhang et al. reported higher *HIF1α* expression in placental shares from twins with IUGR compared to normal twins, although no significant correlation with birth weight could be established [33]. These apparent differences could stem from differences in tissue type, study population and study size.

The incidence of childhood cancer is estimated to increase but the causes remain uncertain [34, 35]. Insults to the genome in the perinatal period are likely to contribute to carcinogenesis and may be more important relative to other life stages because of the higher probability that mutated and genomically unstable cells could populate the rapidly growing tissues of an infant [36]. Mutations of the genome may have particularly adverse consequences in early life, including developmental defects and immune system dysfunction [36, 37]. Gene expression related to DNA damage and immune response among children is observed to correlate with micronuclei frequency (MN) as a consequence of exposure to environmental pollutants [38, 39]. As for RAD52, this is the first time that expression of this DNA damage marker was reported to be associated with measures of fetal growth. RAD52 is a key player in DNA doublestrand break repair and homologous recombination. It forms a heptameric ring, catalyses DNA annealing and mediates Rad51-catalysed strand invasion [40]. The process of DNA repair has previously been linked with changes in birth weight, as a genome-wide methylation study in the Norwegian mother and child cohort identified increased levels of X-ray repair cross complementing 3 (XRCC3) methylation to be associated with increased birth weight [41]. They noted an inverse association between DNA damage gene expression (through increased methylation) and birth weight consistent with our findings. Furthermore, a positive association between DNA damage markers and birthweight was recently reported in the Australian DADHI cohort, where genome damage was measured by scoring MN as a biomarker of both chromosome breakage and/or loss; nucleoplasmic bridges as a biomarker of DNA mis-repair and/or telomere end fusions and nuclear buds were measured as biomarker of gene amplification and/or the removal of unresolved DNA repair complexes. Infant birth weight was associated positively with all the aforementioned chromosomal lesions in cord blood from term newborns [42].

To our knowledge, this is the first report investigating the association between expression of the hypoxia-regulation network including *HIF1α* and several of its downstream targets and newborn growth characteristics in term babies from normal pregnancies. We herewith provide evidence that tight regulation of oxygen tension/hypoxia is important also in healthy pregnancies and show that lower expression is associated with lower birth weight and ponderal index. The public health significance of this finding is important as it provides molecular evidence for the explanation of epidemiological studies that low-birth-weight babies have an increased risk for the development of cardiovascular disease in later life.

We did not identify any associations between placental miR-210 expression and fetal growth characteristics, although miR-210 is a target of HIF1α [43] and has been shown to be increased in placental tissue from preeclamptic women [9]. As miR-210 itself is known to target HIF1α and thereby functions in a negative feedback loop, and other mRNAs such as Akt and p53 can also regulate miR-210 expression [44, 45], this could explain why we did not observe an association with fetal growth in our current analyses.

As such, our study involves a large sample size and expression profiling was performed in placental tissue, the main regulator of fetal development. The current findings must be interpreted within the context of its limitations. At this time we cannot make any predictions on the role of the hypoxia-regulating network in postnatal child growth and adult disease risk. As we only collect placentae at birth, we cannot make any conclusions on dynamics of expression throughout pregnancy.

## Conclusions

We observed significant associations between hypoxia-network members *HIF1, RAD52* and miR-20a expression and ponderal index as well as birth weight as measures of fetal growth in term newborns. The inverse relationship between fetal growth and expression of hypoxia-network members *HIF1, RAD52* and miR-20a indicates that this network is important in potential intrauterine insults.

## Abbreviations

AKT: serine-threonine protein kinase
BMI: body mass index
C-section: Caesarian-section
COX-10: cytochrome C oxidase-10
GAPDH: glyceraldehyde-3-phosphate dehydrogenase
HIF1α: hypoxia-inducible factor 1α
IPO8: importin 8
IRC: inter run calibrator
ISCU: iron sulfur-cluster assembly enzyme
IUGR: intrauterine growth restriction
LBW: low birth weight
miR: microRNA
MIQE: minimum information for publication of quantitative real-time PCR experimens
MN: micronuclei
PI: ponderal index
POLR2A: RNA polymerase II subunit A
PTEN: phosphatase and tensin homolog
RNU6: RNA, U6 small nuclear
TP53: tumor protein 53
UBC: ubiquitin C
XRCC3: X-ray repair cross complementing 3
